# NiCo_2_S_4_@NiMoO_4_ Core-Shell Heterostructure Nanotube Arrays Grown on Ni Foam as a Binder-Free Electrode Displayed High Electrochemical Performance with High Capacity

**DOI:** 10.1186/s11671-017-2180-z

**Published:** 2017-06-15

**Authors:** Yan Zhang, Jie Xu, Yayun Zheng, Yingjiu Zhang, Xing Hu, Tingting Xu

**Affiliations:** School of Physical Engineering and Key Laboratory of Material Physics, Ministry of Education, Zhengzhou University, No. 75 Daxue Road, Zhengzhou, 450052 China

**Keywords:** NiCo_2_S_4_@NiMoO_4_, Core-shell, Nanotube arrays, Ni foam, Supercapaitor

## Abstract

Core-shell-structured system has been proved as one of the best architecture for clean energy products owing to its inherited superiorities from both the core and the shell part, which can provide better conductivity and high surface area. Herein, a hierarchical core-shell NiCo_2_S_4_@NiMoO_4_ heterostructure nanotube array on Ni foam (NF) (NiCo_2_S_4_@NiMoO_4_/NF) has been successfully fabricated. Because of its novel heterostructure, the capacitive performance has been enhanced. A specific capacitance up to 2006 F g^-1^ was obtained at a current density of 5 mA cm^-2^, which was far higher than that of pristine NiCo_2_S_4_ nanotube arrays (about 1264 F g^-1^). More importantly, NiCo_2_S_4_@NiMoO_4_/NF and active carbon (AC) were congregated as positive electrode and negative electrode in an asymmetric supercapacitor. As-fabricated NiCo_2_S_4_@NiMoO_4_/NF//AC device has a good cyclic behavior with 78% capacitance retention over 2000 cycles, and exhibits a high energy density of 21.4 Wh kg^-1^ and power density of 58 W kg^-1^ at 2 mA cm^-2^. As displayed, the NiCo_2_S_4_@NiMoO_4_/NF core-shell herterostructure holds great promise for supercapacitors in energy storage.

**Electronic supplementary material:**

The online version of this article (doi:10.1186/s11671-017-2180-z) contains supplementary material, which is available to authorized users.

## Background

The ever increasing amount of energy consumption has motivated exploration of high-performance clean renewable energy [1–6]. Supercapacitors, considered as the promising dependable devices for energy storage, display excellent power density, rapid charge/discharge properties, long cycling stability, and environmental friendliness, which have received lots of attention from the researchers [7, 8]. At present, supercapacitors use high-surface-area carbon materials to store charge purely by electrostatic in nature (non-Faradaic electric double layers) [9], including carbon nanotube, graphene, and activated carbon. Capitalizing on Faradaic redox reactions, transition metal oxides, metal sulfides, or conducting polymers as pseudocapacitor electrode materials show higher specific capacitances than those carbonaceous electrode materials [2, 10]. Transition metal oxides have several advantages over other pseudocapacitive materials owning the properties of low toxicity, low cost, and natural abundance [11]. Among these transition metal oxides studied so far, ternary metal oxides, like NiCo_2_O_4_ [12], CuCo_2_O_4_ [13], NiMoO_4_ [14], CoMoO_4_ [15], and so on, can provide much higher electrical conductivity and richer electrochemical active sites than their single components, and they have been widely studied in the electrochemical energy field [12–15]. Although great progress has been made on ternary metal oxides electrodes to improve their electrochemical performance, these electrode materials still suffer from insufficient conductivity, slow ion diffusion rates, and serious volume change during the electrochemical procedure, which limit their further application for improving the performance of supercapacitors [16, 17]. Thus, it is vital to explore high-performance novel electrode materials to fulfill the increasing need for the electrochemical energy storage devices.

Lately, numerous attempts have been conducted to develop transition metal sulfides including CoS [18], NiS [19], CuS [20], Co_9_S_8_ [21], and NiCo_2_S_4_ [22] as supercapacitor electrode materials because of the gratifying electrical conductivity in comparison with the corresponding metal oxides [5]. Moreover, the ternary sulfides also can possess a higher conductivity and offer more richer redox reactions than those bare binary sulfides owing to the combine contributions from both metal ions [23, 24]. And NiCo_2_S_4_ as electrode has excellent electrochemical performance in energy devices [23–25]. However, many previous reports still demonstrate that most of the NiCo_2_S_4_ electrodes could not meet the requirement of high capacitance [26]. To address this issue, one possible solution is to design and synthesis different morphologies of metal sulfides with a large electrochemical active surface to enhance the electrochemical behavior. In particular, the core-shell heterostructure nanoarrays exhibit an efficient approach to improve the electrochemical behavior because it can provide many advantages such as the enlarged surface area, the increased conductivity and the synergistic effects produced by the core and shell materials [27].

Recently, various core-shell hybrid structure configurations have been fabricated such as NiCo_2_S_4_@Ni(OH)_2_ [28], NiCo_2_S_4_@Co(OH)_2_ [29], NiCo_2_O_4_@NiMoO_4_ [30], Co_3_O_4_@NiMoO_4_ [31], NiMoO_4_@Ni(OH)_2_ [32] and so forth, which have improved the electrochemical performance. Despite this progress, it is still a big challenge to fabricate the core-shell heterostructure with well-defined morphologies by effective and simple methods [33]. To further optimize the performance, the core-shell heterostructure can be directly grown on current collector which could offer good mechanical adhesion and electrical connection between the active materials and the substrates. Then, this configuration would increase the utilization of the active materials and lead to a higher capacitance [34].

Based on the above ideas, a core-shell heterostructure with the outer layer of NiMoO_4_ nanosheets covering the NiCo_2_S_4_ nanotube arrays on Ni foam has been synthesized through a facile hydrothermal process and a heat treatment, which can be used as an advanced binder-free electrode. The as-prepared NiCo_2_S_4_@NiMoO_4_/NF hybrid electrode exhibits a high specific capacitance up to 2006 F g^-1^ which is much higher than that of pristine NiCo_2_S_4_ nanotube arrays (NiCo_2_S_4_/NF) at 5 mA cm^-2^, and a good cyclic performance of 75% capacitance retained over 2000 cycles at 50 mA cm^-2^. Lately, an asymmetric supercapacitor based on NiCo_2_S_4_@NiMo_2_O_4_/NF and AC delivers a wide voltage window of 1.6 V, a maximum energy density of 21.4 Wh kg^-1^, and a good cyclic stability of 78% capacitance retention at 40 mA cm^-2^ over 2000 cycles. The above results imply that the NiCo_2_S_4_@NiMoO_4_/NF core-shell heterostructure is a promising electrode material in supercapacitor applications.

## Methods

### Synthesis of NiCo_2_S_4_/NF

The NiCo_2_S_4_/NF was fabricated through a two-step hydrothermal process similar to the previous reports [7, 26, 28]. Firstly, the Ni foam (1 × 4 cm) was cleaned in the HCl solution (3 mol L^-1^) and acetone then washed thoroughly using deionized (DI) water and ethanol. The pre-treated Ni foam was obtained. Second, Co(NO_3_)_2_ · 6H_2_O, Ni(NO_3_)_2_ · 6H_2_O and urea were dissolved in 70 mL DI water with a molar ration of 2:1:5. Then the system was moved in a Teflon-lined autoclave with the presence of cleaned Ni foam. After maintaining at 120 °C for 12 h, the Ni-Co precursor was successfully prepared. The NiCo_2_S_4_/NF was obtained by treating the Ni-Co precursor with Na_2_S solution (0.03 mol L^-1^) under the 90 °C for 12 h through an ion-exchange process. The average mass loading of as-prepared NiCo_2_S_4_/NF was around 2 mg cm^-2^.

### Synthesis of NiCo_2_S_4_@NiMoO_4_/NF

The NiCo_2_S_4_@NiMoO_4_/NF was prepared by a hydrothermal route combining with a calcination process were according to previously published works with some modified [32, 35]. Typically, the NiCo_2_S_4_/NF was put into the 70 mL solution containing 1 mmol Ni(NO_3_)_2_ · 6H_2_O and 1 mmol Na_2_MoO_4_ · 2H_2_O through a hydrothermal treatment under 100 °C for 4 h. Therein, the as-obtained sample was annealed by keeping the temperature at 400 °C for 2 h under Ar atmosphere. The mass loading of NiCo_2_S_4_@NiMoO_4_ was about 3 mg cm^-2^.

### Material Characterization

The structure of the prepared materials was investigated using X-ray diffraction (XRD, Netherlands Philip X’ Pert). The information of morphologies from the NiCo_2_S_4_/NF and NiCo_2_S_4_@NiMoO_4_/NF was studied by scanning electron microscope (SEM, JSM-6700F, JEOL) and transmission electron microscope (TEM, JEM-2100, 200 kV, JEOL). X-ray photo-electron spectroscopy (XPS) measurements were conducted on Thermo Scientific ESCALAB 250XI spectrometer.

### Electrochemical measurements

The three-electrode configuration was conducted on the electrochemical workstation (CS 2350, Wuhan) to analyze the electrochemical properties in 2 mol L^-1^ KOH electrolyte. The working electrode was NiCo_2_S_4_/NF and NiCo_2_S_4_@NiMoO_4_/NF (1× 1 cm in area), the Pt foil was employed as the counter electrode and standard calomel electrode (SCE) was acted as the reference electrode. Techniques contained cyclic voltammetry (CV), galvanostatic charge-discharge (GCD) and electrochemical impedance spectroscopy (EIS). The EIS tests were conducted with the frequency of 0.01 Hz~100 kHz and a superimposed sinusoidal voltage of 5 mV amplitude. Based on the discharge curves, the specific capacitances (C_s_, F g^-1^) were calculated on the basis of the following equation: C_s_ = IΔt/mΔV, where m (g), I (A), ΔV (V) and Δt (s) represent the mass, current, voltage window, and the time during the discharge procedure, respectively.

### Fabrication of the Asymmetric Supercapacitor

Electrochemical measurements of the asymmetric supercapacitor (ASC) device were investigated in a two-electrode configuration. The configuration took NiCo_2_S_4_@NiMoO_4_/NF and AC as the positive and negative electrode, respectively, a filter paper as separator. Then, we wrapped them with the tape for packaging. Afterwards, we immersed them in the electrolyte of 2 mol L^-1^ KOH and obtained the final assembled asymmetric NiCo_2_S_4_@NiMoO_4_//AC device (Additional file 1: Figure S1). Particularly, the active carbon was mixed with 10 wt% acetylene black and 5 wt% polyvinylidene fluoride (PVDF) to form the slurry to prepare the AC electrode. Subsequently, the slurry was directly coated onto the pre-treated Ni foam (1 × 1 cm in area) and dried in vacuum at 60 °C for 12 h. The mass of the positive and negative electrodes were determined with the balance theory of Q_+_ = Q_-_ (Q = C_s_mΔV) to ensure an efficient charge storage, where C_s_ (F g^-1^), m (g) and ΔV (V) stand for the specific capacitance, mass of the electrode and the potential window, respectively. Based on the above balance theory, the optimal mass loading of the negative electrode of AC is about 24.84 mg cm^-2^.

## Results and Discussion

**Fig. 1:**
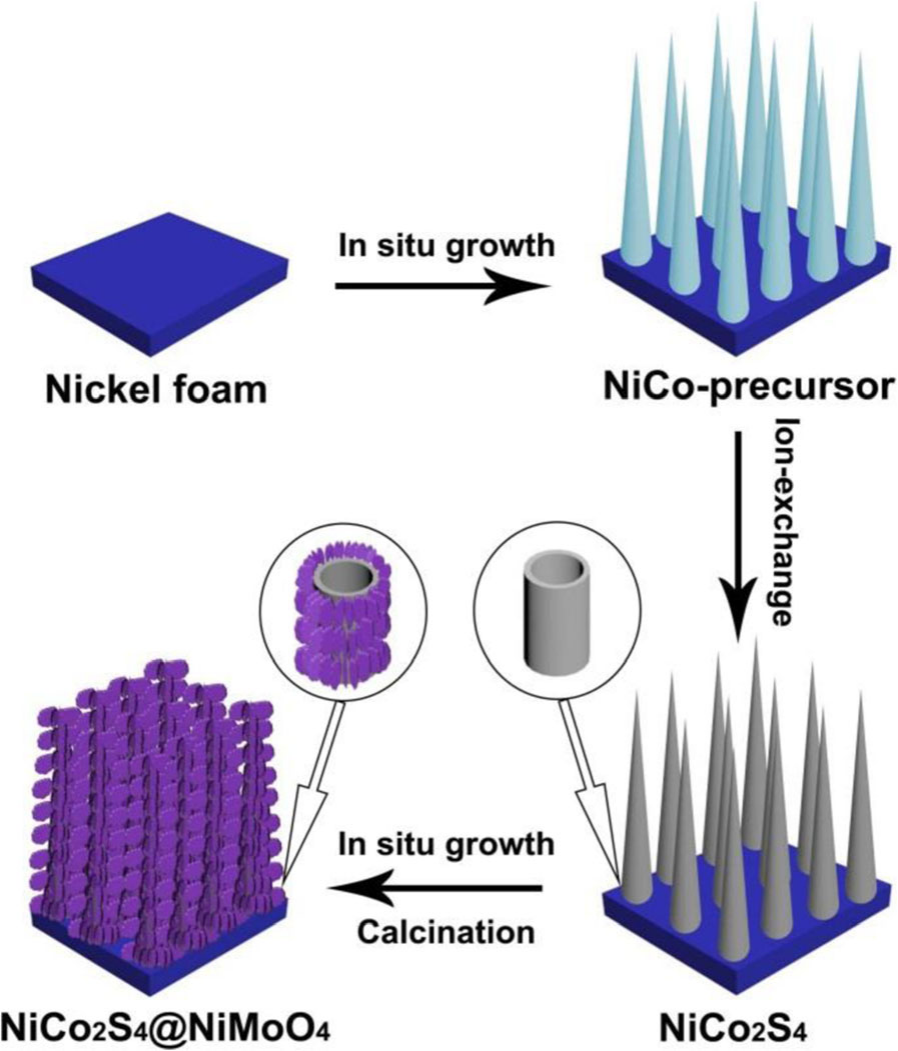
Schematic fabrication process of NiCo_2_S_4_@NiMoO_4_/NF

The fabrication process of the hierarchical NiCo_2_S_4_@NiMoO_4_/NF is displayed in Fig. 1. Initially, under a two-step hydrothermal method which contains an in situ growth procedure and an ion-exchange process, the NiCo_2_S_4_ nanotube arrays on highly conductive microporous Ni foam were obtained. Subsequently, NiMoO_4_ interconnected nanosheets shell was deposited on the backbone of NiCo_2_S_4_ nanotube arrays through a hydrothermal treatment as well as an annealing process.

**Fig. 2:**
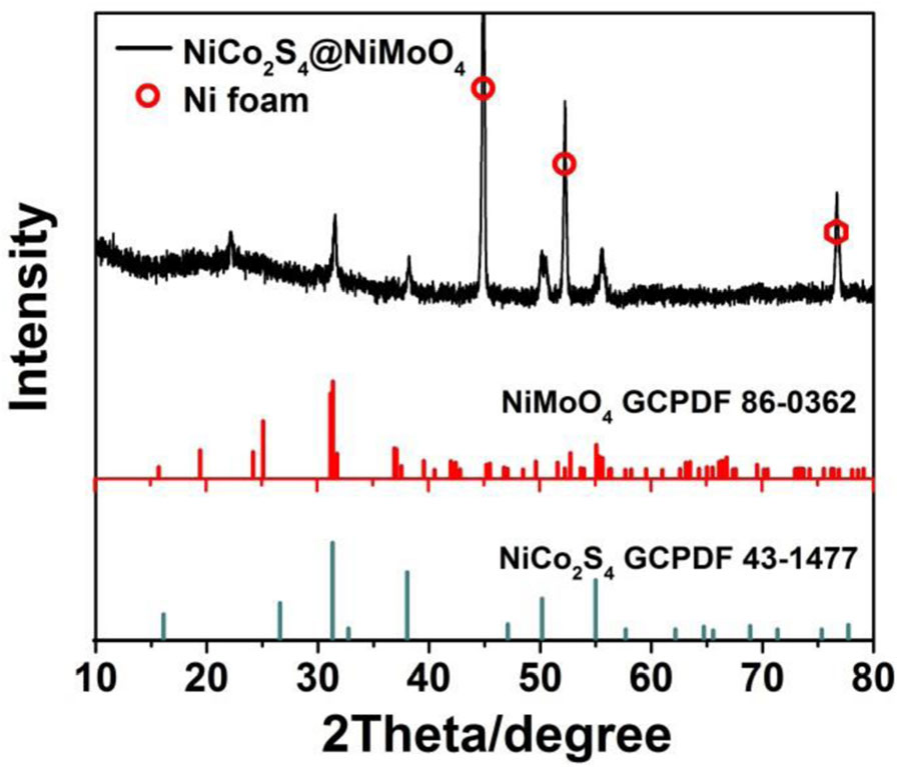
XRD pattern for NiCo_2_S_4_@NiMoO_4_/NF

The XRD pattern of as-prepared NiCo_2_S_4_@NiMoO_4_ core-shell nanotube arrays on Ni foam is shown in Fig. 2. The substrate of Ni foam corresponds to three main peaks in the pattern. Several other strong peaks of 31.7°, 38.2°, 50.4°, and 55.5° can be well indexed to NiCo_2_S_4_ (PDF cards No. 43-1477), and the diffraction peaks of 31.4°, 36.9°, and 55.1° belong to NiMoO_4_ (PDF cards No. 86-0362), which indicate the formation of the NiCo_2_S_4_ and NiMoO_4_. Besides, the XPS results of the as-prepared NiCo_2_S_4_@NiMoO_4_ are shown in Additional file 1: Figure S2. The full survey spectrum mainly displays that presence of the Ni 2p, Co 2p, Mo 3d, S 2p, O 1 s in the product (Additional file 1: Figure S2A). The binding energies of Ni 2p and Co 2p are in accordance with the formation of NiCo_2_S_4_ [36, 37]. The XPS results as shown in Additional file 1: Figure S2 display that the composite contains Ni^2+^, Ni^3+^, Co^2+^, Co^3+^ and Mo^6+^, which are agree with the phase structure of NiCo_2_S_4_@NiMoO_4_ [36, 38, 39].

**Fig. 3:**
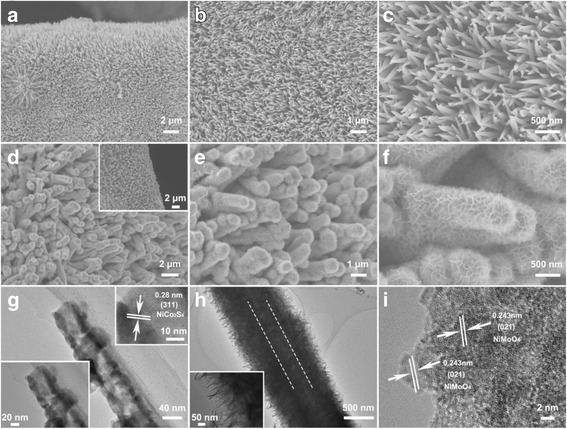
SEM images for NiCo_2_S_4_/NF (**a**–**c**) and NiCo_2_S_4_@NiMoO_4_/NF (**d**–**f**) at different magnifications. **g** TEM images of an individual NiCo_2_S_4_ nanotube detached from Ni foam; the above *inset* is the corresponding HRTEM image of a single nanotube. **h** TEM images and **i** HRTEM images of an individual NiCo_2_S_4_@NiMoO_4_ core-shell structure

The general morphologies and microstructure of the NiCo_2_S_4_/NF and NiCo_2_S_4_@NiMoO_4_/NF electrode materials are presented in Fig. 3. The SEM images at different magnifications of the NiCo_2_S_4_ nanotubes on Ni foam are displayed in Fig. 3a–c. From the images in Fig. 3a and b, a grass-like three-dimensional (3D) nanostructure homogeneously covered on the substrate of Ni foam was formed by a large number of NiCo_2_S_4_ nanotubes. And, the diameter of the nanotube is approximately 70–100 nm (Fig. 3c). Afterward, the surface of NiCo_2_S_4_ nanotubes turns rough, and a layer shell of NiMoO_4_ interconnecting nanosheets is fully deposited on the surface of NiCo_2_S_4_ nanotubes, which results in a hierarchical core-shell heterostructure (as shown in Fig. 3d–f). The obtained NiCo_2_S_4_@NiMoO_4_ nanotubes are well aligned on Ni foam skeletons in large-scale (Fig. 3d and inset). The higher magnification SEM images (Fig. 3e and f) reveal that the NiMoO_4_ nanosheets are crossed-linked with each other and filling both the surface of the NiCo_2_S_4_ nanotubes and the spaces between them. Therefore, a high specific-surface-area construction has been generated and the NiCo_2_S_4_@NiMoO_4_ nanotubes have an average diameter around 700 nm. The detailed structure of NiCo_2_S_4_/NF and NiCo_2_S_4_@NiMoO_4_/NF is further provided by TEM. Figure 3g exhibits the TEM images of NiCo_2_S_4_ nanotubes scraped from Ni foam. The image shows that the NiCo_2_S_4_ nanotubes have a clear hollow nanostructure. The magnified image inset in Fig. 3g at lower left shows that the NiCo_2_S_4_ nanotube displays the shell thickness of 15 ± 2 nm. The inset at the upper right further confirmed the formation of NiCo_2_S_4_ with a lattice spacing of 0.28 nm in according with the (311) plane of cubic phase. The TEM images (Fig. 3h) of NiCo_2_S_4_@NiMoO_4_/NF confirm that the NiMoO_4_ nanosheets are uniformly covered on the surface of NiCo_2_S_4_ nanotubes, and the thickness of NiMoO_4_ shell is around 300 nm which is consistent with the SEM images. Figure 3h inset clearly exhibits the layer containing a large number of thin nanosheets full of stack and folds which is benefit to the ion diffusion during the electrochemical reaction. HRTEM (High Resolution Transmission Electron Microscopy) image shows the lattice fringes of 0.243 nm are matched well with the (021) plane of the NiMoO_4_ layer (Fig. 3i). The above results demonstrate the NiCo_2_S_4_@NiMoO_4_ core-shell nanotubes have been built which is in accordance with the XRD patterns.

**Fig. 4:**
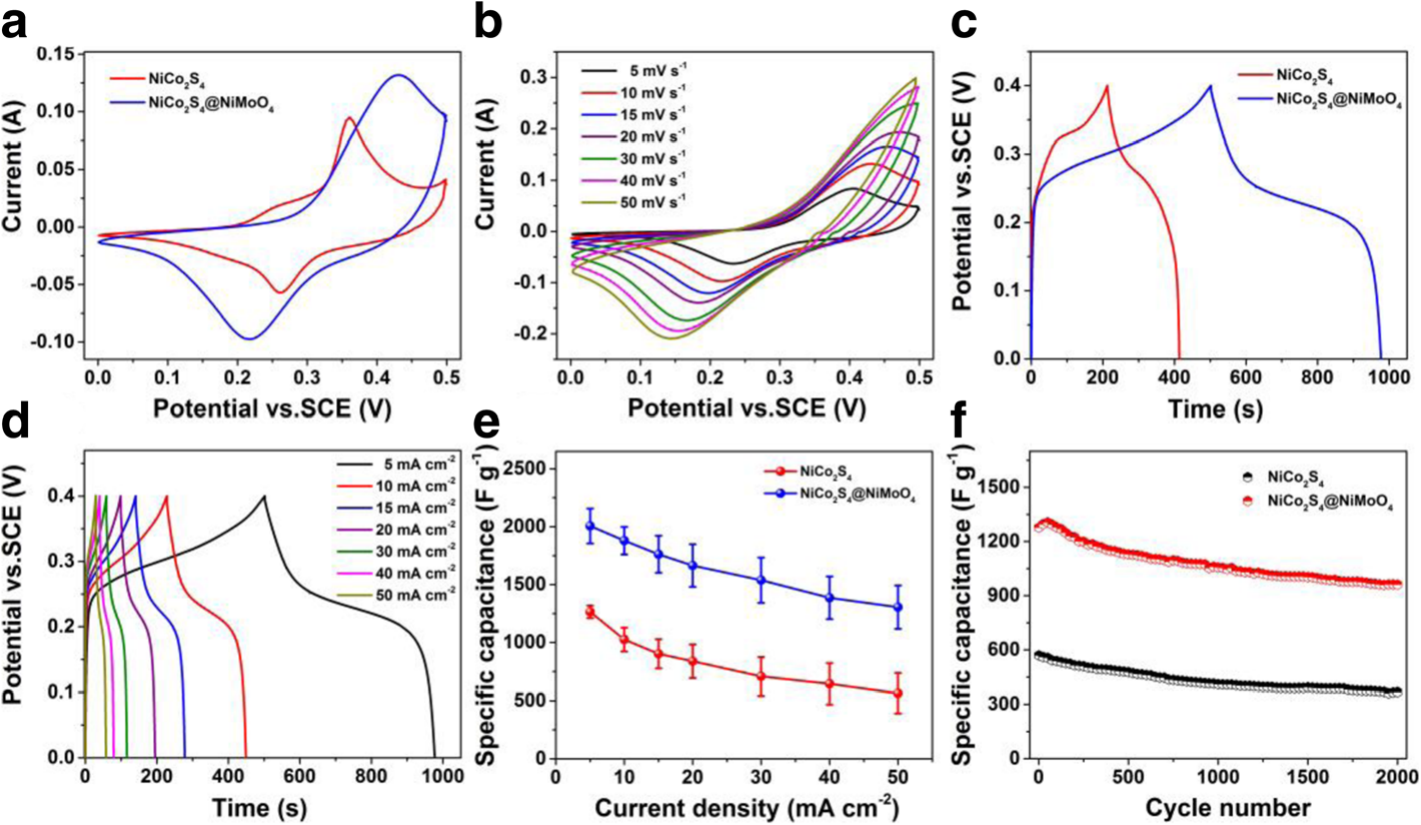
**a** The comparison of the CV curves of NiCo_2_S_4_, NiCo_2_S_4_@NiMoO_4_ at the scan rate of 10 mV s^-2^. **b** CV curves of the NiCo_2_S_4_@NiMoO_4_ product at the scan rates of 5, 10, 15, 20, 30, 40, 50 mV s^-1^. **c** Comparison of GCD curves of the NiCo_2_S_4_, NiCo_2_S_4_@NiMoO_4_ at a current density of 5 mA cm^-2^. **d** GCD curves of the NiCo_2_S_4_@NiMoO_4_ composite at the current densities of 5, 10, 15, 20, 30, 40, 50 mA cm^-2^. **e** Specific capacitance of the NiCo_2_S_4_, NiCo_2_S_4_@NiMoO_4_ composite at different current densities. **f** Cycling performance of NiCo_2_S_4_, NiCo_2_S_4_@NiMoO_4_ composite at 50 mA cm^-2^ for 2000 cycles

The electrochemical performance of NiCo_2_S_4_/NF and NiCo_2_S_4_@NiMoO_4_/NF binder-free electrodes were studied in a three-electrode configuration by measuring techniques of CV, GCD and EIS (Fig. 4, Additional file 1: Figure S3 and S4). Figure 4a exhibits the CV curves of NiCo_2_S_4_/NF electrode and NiCo_2_S_4_@NiMoO_4_/NF electrode with a potential window of 0–0.5 V at 10 mV s^-1^. For the NiCo_2_S_4_/NF electrode, a couple of redox peaks are visible, which are mainly coming from the redox reactions in regard to the M^2+^/M^3+^ (M = Ni, Co) redox couples [28], demonstrating the typical pseudocapacitive performance. For the NiCo_2_S_4_@NiMoO_4_/NF electrode, the expanded peaks are due to the M^2+^/M^3+^ (M = Ni, Co) redox couples from the NiCo_2_S_4_ core and the Ni^2+^/Ni^3+^ redox couples of the NiMoO_4_ shell. During the electrochemical process, the redox reaction of Mo atom does not occur. Then, the redox behavior of Mo has no contribution to the tested capacitance [32]. The Mo element played a key role is to improve the conductivity of the ternary metal oxides and to gain the enhanced electrochemical performance [6]. The capacitances of the electrode are represented by the areas surrounded by the CV curves. Compared with the NiCo_2_S_4_/NF, the NiCo_2_S_4_@NiMoO_4_/NF electrode owned an enlarged area by the presence of NiMoO_4_ nanosheets, revealing the hybrid core-shell electrode possesses a higher specific capacitance. The CV curves of NiCo_2_S_4_@NiMoO_4_/NF and NiCo_2_S_4_/NF electrode at various scan rates are showed in Fig. 4b and Additional file 1: Figure S3A, respectively. The shapes of the curves and the presence of the redox peaks both demonstrate the pseudocapacitive nature of the electrode. As the scan rate increased, the shape of all the curves is still maintained with a little shift of the peaks position owing to the polarization behavior of the electrodes [35]. The GCD measurement determines the capacitive property of NiCo_2_S_4_/NF electrode and NiCo_2_S_4_@NiMoO_4_/NF hybrid electrode. Compared with the pristine NiCo_2_S_4_, the NiCo_2_S_4_@NiMoO_4_ could store more charges due to it delivers a longer discharging time at 5 mA cm^-2^ (Fig. 4c). Besides, in each curve, there is a distinct voltage plateau existing in the charge/discharge process, which reveals the capacitance characteristics generating from the redox reactions, which is consistent with the CV curves. Figure 4d and Additional file 1: Figure S3B display the GCD curves of the prepared electrodes at different current densities. There is a distinct plateau region in every curve proving the pseudocapacitive performance of electrodes. Figure 4e shows the specific capacitances at various current densities of the prepared two electrodes. The specific capacitance of the bare NiCo_2_S_4_ was calculated to be 1264, 1025, 903, 838, 708, 645, 572 F g^-1^ at 5, 10, 15, 20, 30, 40, 50 mA cm^-2^, respectively. In contrast with the bare NiCo_2_S_4_, the NiCo_2_S_4_@NiMoO_4_ displays the significantly enhanced specific capacitances as high as 2006, 1879, 1761, 1664, 1538, 1386, 1305 F g^-1^ at the current densities of 5, 10, 15, 20, 30, 40, 50 mA cm^-2^, respectively. The hybrid electrode possesses a higher capacity mainly due to the five merits as follows: (1) The designed core-shell hybrid configuration and the microporous feature for 3D Ni foam facilitate the diffusion of the electrolyte ions. (2) For redox reactions, the nanotube arrays could result in more exposed electroactive sites. (3) The porous NiCo_2_S_4_ skeleton with high conductivity builds the electrical conductive pathways for active materials leading to the enhanced conductivity and a fast reversible redox reaction. (4) The binder-free characteristic of the NiCo_2_S_4_@NiMoO_4_ enables a low interfacial resistance and the absence of addictive would greatly reduce the “inactive” surface in the electrode [26, 40]. (5) The synergistic effect of the NiCo_2_S_4_ nanotubes core and NiMoO_4_ nanosheets shell also provides a positive effect on the capacitance. Based on the calculated capacitive results showed in Fig. 4e, the capacitance of NiCo_2_S_4_@NiMoO_4_ remains around 65.1% with the increasing of current density, which is higher than the pristine NiCo_2_S_4_ (45.3%). Therefore, the good rate capability is not only owing to the higher conductivity of the NiCo_2_S_4_, but also due to the highly porous structure of the interconnected NiMoO_4_ nanosheets filled both on the surface of the NiCo_2_S_4_ nanotubes as well as the spaces between them, which further increases the accessibility of the microscopic area.

The cyclic performance plays an important role in supercapacitor devices. Figure 4f shows the cycling stabilities of the NiCo_2_S_4_ and NiCo_2_S_4_@NiMoO_4_ hybrid electrodes after 2000 cycles at 50 mA cm^-2^. With the cycle number increasing, the specific capacitance gradually decreases. Over 2000 cycles, there is still 75.3% of its initial capacitance retained and it performs better than NiCo_2_S_4_ (64.6% over 2000 cycles). For NiCo_2_S_4_@NiMoO_4_ electrode, the specific capacitance increases at the initial 100 cycles, which is because the electrode activation increases the available active sites [41]. Besides, EIS measurement was carried out to further examine the excellent electrochemical performance of the NiCo_2_S_4_@NiMoO_4_ electrode. Additional file 1: Figure S4 displays the impedance Nyquist plots of the NiCo_2_S_4_@NiMoO_4_ hybrid electrode before and after 2000 cycles. The Nyquist plots were similar to each other which contained a quasi-semicircle in the high frequency region and a straight line in the low frequency region. The straight line in the low frequency region shows the Warburg resistance which is ascribed to the diffusion behavior of the electrolyte to the electrode surface [42, 43]. And the Warburg resistances of the hybrid electrode before and after cycling are almost unchanged, indicating the good cyclic stability of this electrode. And this is in accordance with the electrochemical performance analyzed above.

**Fig. 5:**
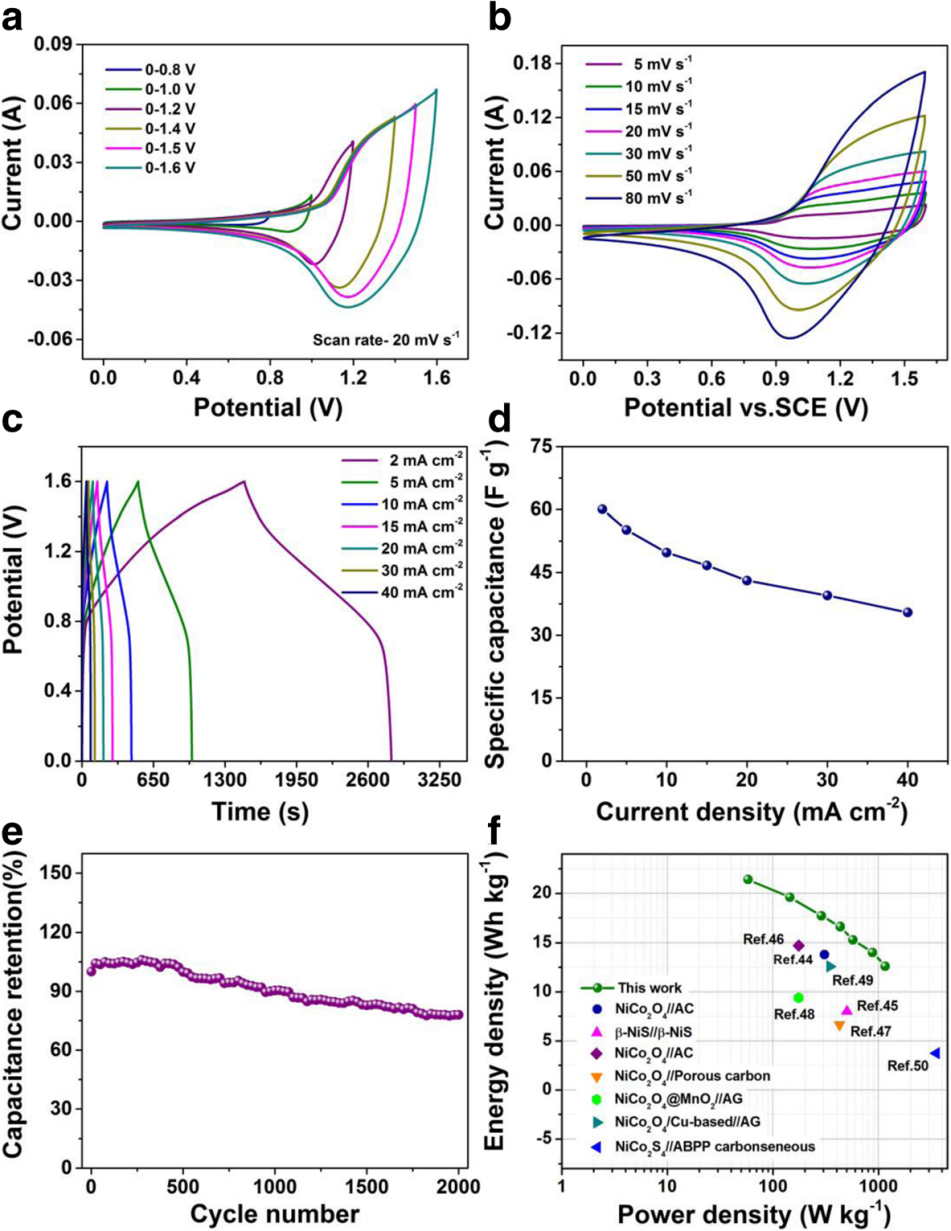
**a** CV curves of NiCo_2_S_4_@NiMoO_4_//AC asymmetric supercapacitor collected in different voltage windows at 20 mV s^-1^. **b** CV curves of NiCo_2_S_4_@NiMoO_4_//AC at different scan rates. **c** GCD curves of NiCo_2_S_4_@NiMoO_4_//AC at different current densities. **d** Specific capacitances of NiCo_2_S_4_@NiMoO_4_//AC at different current densities. **e** Cycling performance of NiCo_2_S_4_@NiMoO_4_//AC at 40 mA cm^-2^. **f** Ragone plots of energy density and power density of NiCo_2_S_4_@NiMoO_4_//AC

To value the potential application of NiCo_2_S_4_@NiMoO_4_ electrode in supercapacitors, an asymmetric supercapacitor device in a two-electrode configuration was constructed with the NiCo_2_S_4_@NiMoO_4_ and AC electrode act as the positive electrode and negative electrode with the area of 1 cm^2^, respectively, a filter paper as the separator and 2 mol L^-1^ KOH as the electrolyte. The specific capacitance of active carbon is 85.07 F g^-1^ at a current density of 5 A g^-1^ (Additional file 1: Figure S5). Figure 5a shows the CV curves of the device at various voltage windows from 0–0.8 to 0–1.6 V. From the image we obtained the voltage window of the ASC device can achieve 1.6 V as expected. The CV curves of the device at different scan rates are shown in Fig. 5b. The shapes of the CV curves at various scan rates are almost maintained, revealing the excellent capacitance behavior of the ASC device. GCD curves of the NiCo_2_S_4_@NiMoO_4_//AC device from 2 to 40 mA cm^-2^ in the potential window of 0–1.6 V are further illustrated in Fig. 5c. The specific capacitance evaluated from the discharging curves are 60.05, 55.16, 49.74, 46.66, 43.06, 39.50, and 35.45 F g^-1^ at 2, 5,10, 15, 20, 30, and 40 mA cm^-2^, respectively, as exhibited in Fig. 5d. The cycling life of the capacitor has been measured by the virtue of GCD cycling at 40 mA cm^-2^ (Fig. 5e). After 2000 cycles, the specific capacitance remains 78%, demonstrating its good cycle stability. The Impedance Nyquist plots of the NiCo_2_S_4_@NiMoO_4_//AC device before and after 2000 cycles have been shown in Additional file 1: Figure S6. The plots show that the Warburg resistances of the device are almost have no change before and after cycling, demonstrating the good stability of the asymmetric device. Figure 5f displays the relations between the energy density and power density in contrast with other devices. The NiCo_2_S_4_@NiMoO_4_//AC device displays 21.4 Wh kg^-1^ at 58 W kg^-1^, and still maintains 12.6 Wh kg^-1^ at a power density of 1158 W kg^-1^. As compared to previous reported publications, the energy density of our work is higher than those of NiCo_2_O_4_//AC (13.8 Wh kg^-1^) [44], β-NiS//β-NiS (7.97 Wh kg^-1^) [45], NiCo_2_O_4_//AC (14.7 Wh kg^-1^) [46], NiCo_2_O_4_// Porous carbon (6.61 Wh kg^-1^) [47], NiCo_2_O_4_@MnO_2_//AG (activated graphenes) (9.4 Wh kg^-1^) [48], NiCo_2_O_4_/Cu-based//AG (12.6 Wh kg^-1^) [49], NiCo_2_S_4_//ABPP (activated balsam pear pulp) carbonceneous (3.72 Wh kg^-1^) [50].

## Conclusions

In short, novel hierarchical NiCo_2_S_4_@NiMoO_4_ nanotube arrays with the core-shell heterostructure have been successfully deposited on Ni foam. As the electrode for supercapacitors, it displays a high specific capacitance of 2006 F g^-1^ at 5 mA cm^-2^ and a good cyclic stability (75% after 2000 cycles at 50 mA cm^-2^). Moreover, an asymmetric supercapacitor has been obtained based on NiCo_2_S_4_@NiMoO_4_ and AC as the positive and negative electrode, respectively, which achieves a specific capacitance of 60.05 F g^-1^ at 2 mA cm^-2^ with a potential window of 1.6 V. It also delivers a maximum energy density of 21.4 Wh kg^-1^ and a good cyclic stability (78% over 2000 cycles at 40 mA cm^-2^), which make it a promising candidate in the field of supercapacitors.

## Additional file

Additional file 1: Supporting information. **Figure S1.** Schematic illustration (A) and photograph (B) of the as-fabricated NiCo_2_S_4_@NiMoO_4_//AC device. **Figure S2.** XPS spectra of the (A) survey spectrum, (B) Ni 2p, (C) Co 2p, (D) Mo 3d, (E) S 2p and (F) O 1 s of the NiCo_2_S_4_@NiMoO_4_ composite. **Figure S3.** (A) CV curves at different scan rates and (B) GCD curves at different current densities of NiCo_2_S_4_. **Figure S4.** Impedance Nyquist plots of the NiCo_2_S_4_@NiMoO_4_ hybrid electrode before and after 2000 cycles in a three-electrode system. **Figure S5.** CV curves of the AC electrode at different scan rates (A), GCD curves of the AC electrode at different current densities (B), the specific capacitance change of the AC electrode at different current densities (C). **Figure S6.** Impedance Nyquist plots of the NiCo_2_S_4_@NiMoO_4_//AC device before and after 2000 cycles. (DOCX 2512 kb)

## Abbreviations

ABPP: Activated balsam pear pulp
AC: Active carbon
AG: Activated graphenes
ASC: Asymmetric supercapacitor
CV: Cyclic voltammetry
DI: Deionized
EIS: Electrochemical impedance spectroscopy
GCD: Galvanostatic charge-discharge
HRTEM: High-resolution transmission electron microscopy
NF: Ni foam
PVDF: Polyvinylidene fluoride
SCE: Standard calomel electrode
SEM: Scanning electron microscope
TEM: Transmission electron microscope
XPS: X-ray photo-electron spectroscopy
XRD: X-ray diffraction
