# Supplementary material for: NiCo2S4@NiMoO4 Core-Shell Heterostructure Nanotube Arrays Grown on Ni Foam as a Binder-Free Electrode Displayed High Electrochemical Performance with High Capacity
Source: Nanoscale Res Lett. 2017 Jun 15;12:412. doi: 10.1186/s11671-017-2180-z (PMC5472642; doi:10.1186/s11671-017-2180-z)
Supplement: Additional file 1: — Supporting information. Figure S1. Schematic illustration (A) and photograph (B) of the as-fabricated NiCo2S4@NiMoO4//AC device. Figure S2. XPS spectra of the (A) survey spectrum, (B) Ni 2p, (C) Co 2p, (D) Mo 3d, (E) S 2p and (F) O 1 s of the NiCo2S4@NiMoO4 composite. Figure S3. (A) CV curves at different scan rates and (B) GCD curves at different current densities of NiCo2S4. Figure S4. Impedance Nyquist plots of the NiCo2S4@NiMoO4 hybrid electrode before and after 2000 cycles in a three-electrode system. Figure S5. CV curves of the AC electrode at different scan rates (A), GCD curves of the AC electrode at different current densities (B), the specific capacitance change of the AC electrode at different current densities (C). Figure S6. Impedance Nyquist plots of the NiCo2S4@NiMoO4//AC device before and after 2000 cycles. (DOCX 2512 kb) [file 11671_2017_2180_MOESM1_ESM.docx]

Support Information

**NiCo_2_S_4_@NiMoO_4_ core-shell heterostructure nanotube arrays grown on Ni foam as a binder-free electrode displayed high electrochemical performance with high capacity**

Yan Zhang ^1^, Jie Xu ^1*^, Yayun Zheng ^1^, Yingjiu Zhang ^1*^, Xing Hu ^1^, Tingting Xu ^1^

*School of Physical Engineering and Key Laboratory of Material Physics, Ministry of Education, Zhengzhou University, NO. 75 Daxue Road, Zhengzhou 450052, China*

^*^ Corresponding author: Tel. +86 371 67766870, Fax. +86 371 67766629

*E-mail address*: xujie@zzu.edu.cn; [zhangyj2006@zzu.edu.cn](mailto:zhangyj2006@zzu.edu.cn)


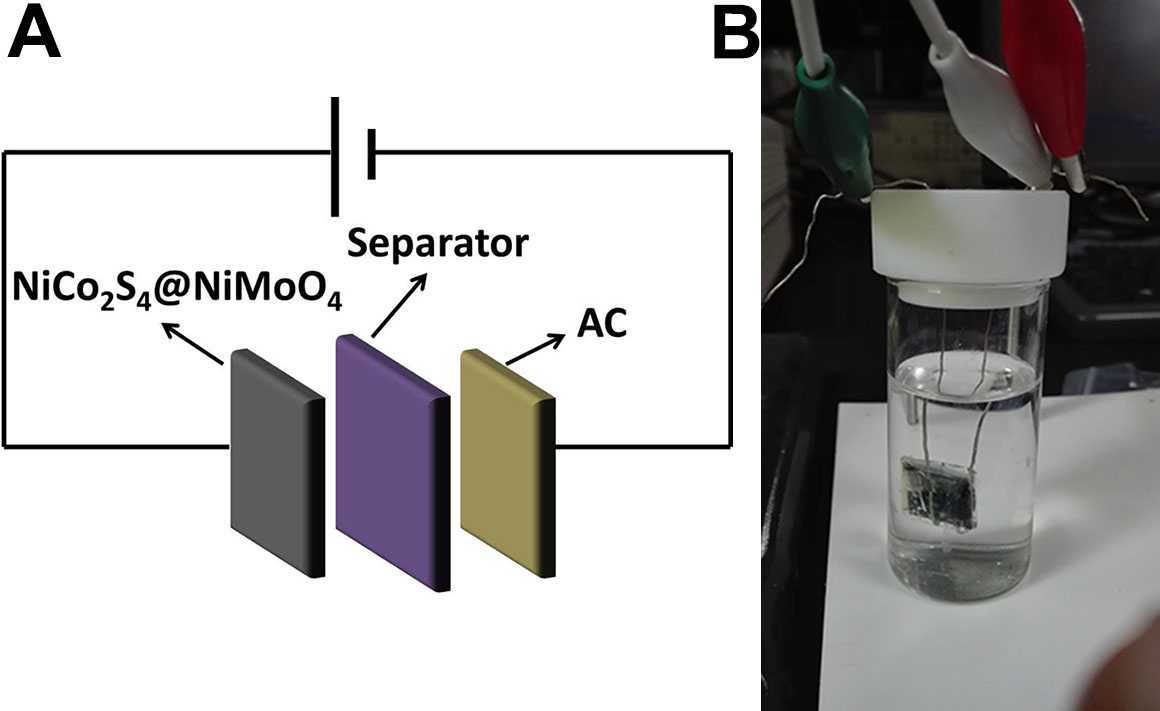


**Fig. S1** Schematic illustration (A) and photograph (B) of the as-fabricated NiCo_2_S_4_@NiMoO_4_//AC device.


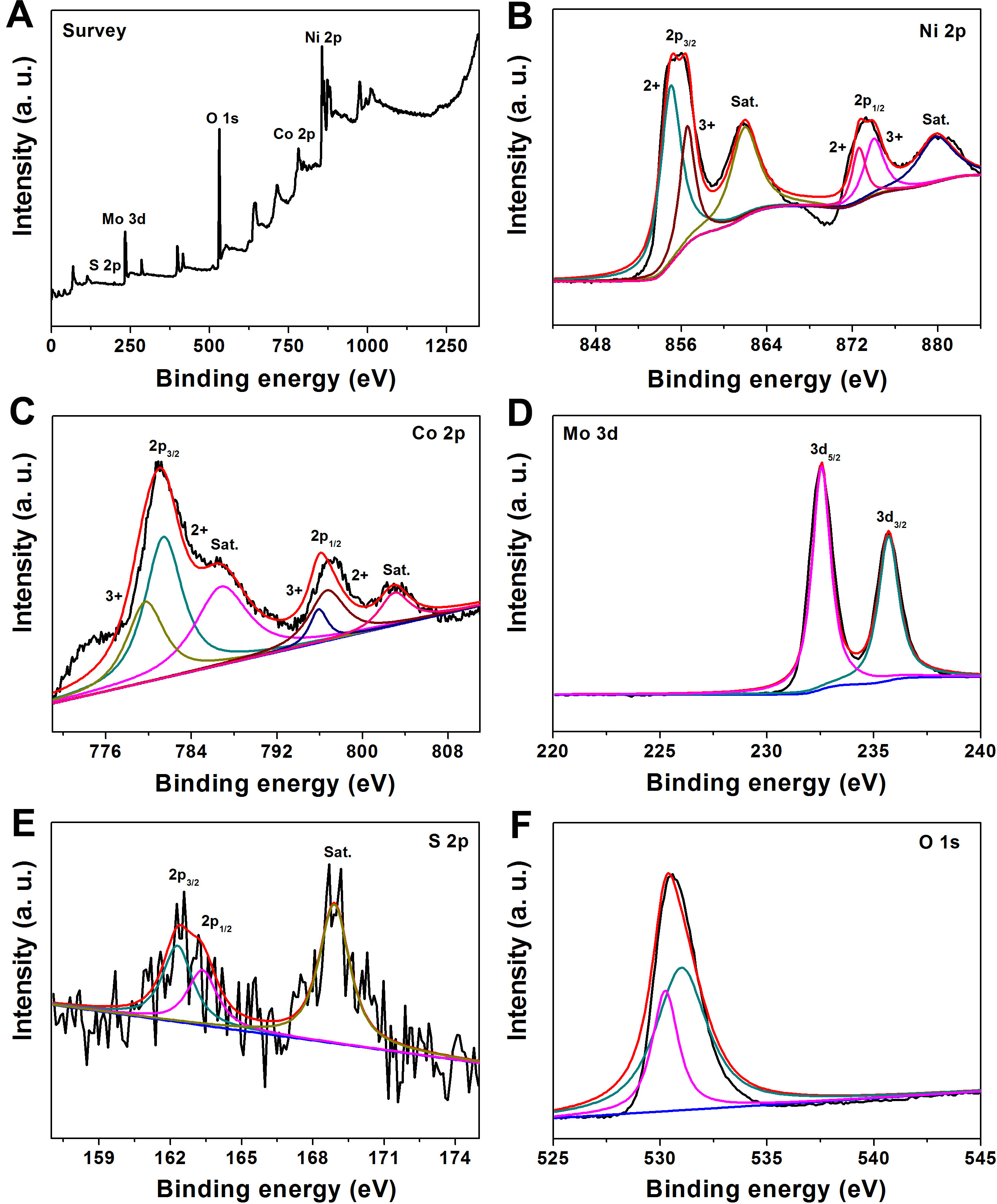


**Fig. S2** XPS spectra of the (A) survey spectrum, (B) Ni 2p, (C) Co 2p, (D) Mo 3d, (E) S 2p and (F) O 1s of the NiCo_2_S_4_@NiMoO_4_ composite.

The surface electronic configuration and elemental compositions of the elements in the composite are investigated by XPS measurements. Then the XPS measurements were conducted based on the powder products which were scraped from Ni foam. And the results have been added in Fig. S2. As expected, the full survey spectrum mainly displays that presence of the Ni 2p, Co 2p, Mo 3d, S 2p, O 1s in the as-prepared NiCo_2_S_4_@NiMoO_4_ (Fig. S2A). It is recognized that, there are different ionic states of Ni and Co existing in the spinel NiCo_2_S_4_. As shown in Fig. S2B, using a Gaussian fitting method, the Ni 2p XPS spectrum was fitted with two spin-orbit doublets (Ni 2p_3/2_ and Ni 2p_1/2_), characteristic of Ni^3+^ and Ni^2+^, and the corresponding shakeup satellites (identified as ‘‘Sat.’’). Similarly, the de-convoluted Co 2p displays the two binding peaks of 781.0 eV and 796.8 eV are correspond to the Co 2p_3/2_ and Co 2p_1/2_, characteristic of Co^2+^ and Co^3+^, along with two shakeup satellites (Fig. S2C). Agreed with the previous reports, the composite contains Ni^3+^, Ni^2+^, Co^2+^ and Co^3+^ due to the existence of NiCo_2_S_4_ core [1]. The Mo 3p XPS spectrum (Fig. S2D) depicts the two major binding energy peaks at 232.6 and 235.7 eV, which can be assigned to the Mo 3p_5/2_ and Mo 3p_3/2_, representing the existence of Mo^6+^ oxidation state [2,3]. The S spectrum is displayed in Fig. S2E. The peaks at 162.5 and 163.2 eV correspond to S 2p_3/2_ and S 2p_1/2_, respectively. In detail, the binding energy at 162.5 eV is may be due to the S^2−^ in low coordination at the surface, while the binding energy at 163.2 eV is attributed to metal-sulfur bonds [4]. Fig. S2F shows the O 1s spectrum and it can be divided into two main peaks. Similarly, the peak at 530.2 eV is coming from the metal-oxygen bonds, and the peak at 531.0 eV can be due to the oxygen ions in low coordination at the surface [2]. The XPS results display that the chemical composition contain Ni^2+^ and Mo^6+^, which are agree with the phase structure of NiMoO_4_. Furthermore, it can confirm the successful formation of NiCo_2_S_4_@NiMoO_4_ composite.


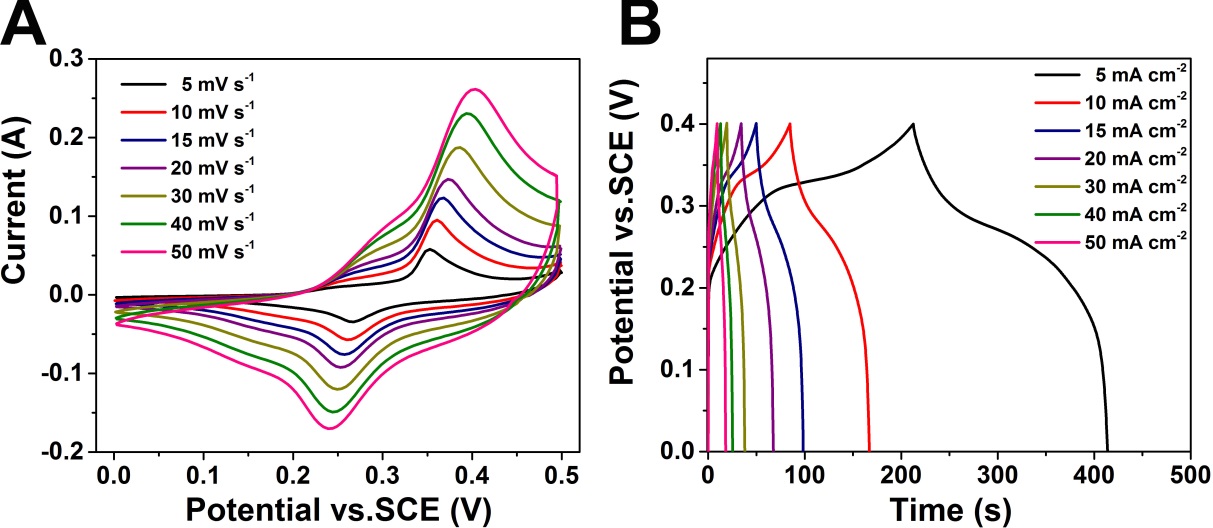


**Fig. S3** (A) CV curves at different scan rates and (B) GCD curves at different current densities of NiCo_2_S_4_.


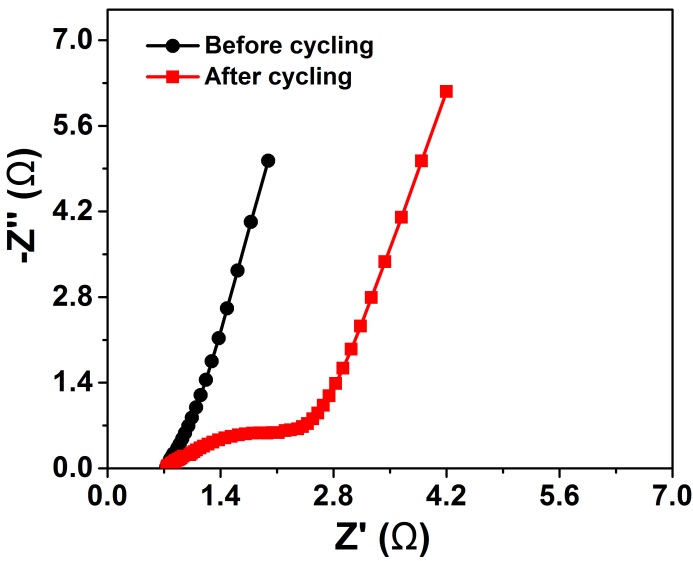


**Fig. S4** Impedance Nyquist plots of the NiCo_2_S_4_@NiMoO_4_ hybrid electrode before and after 2000 cycles in a three-electrode system.


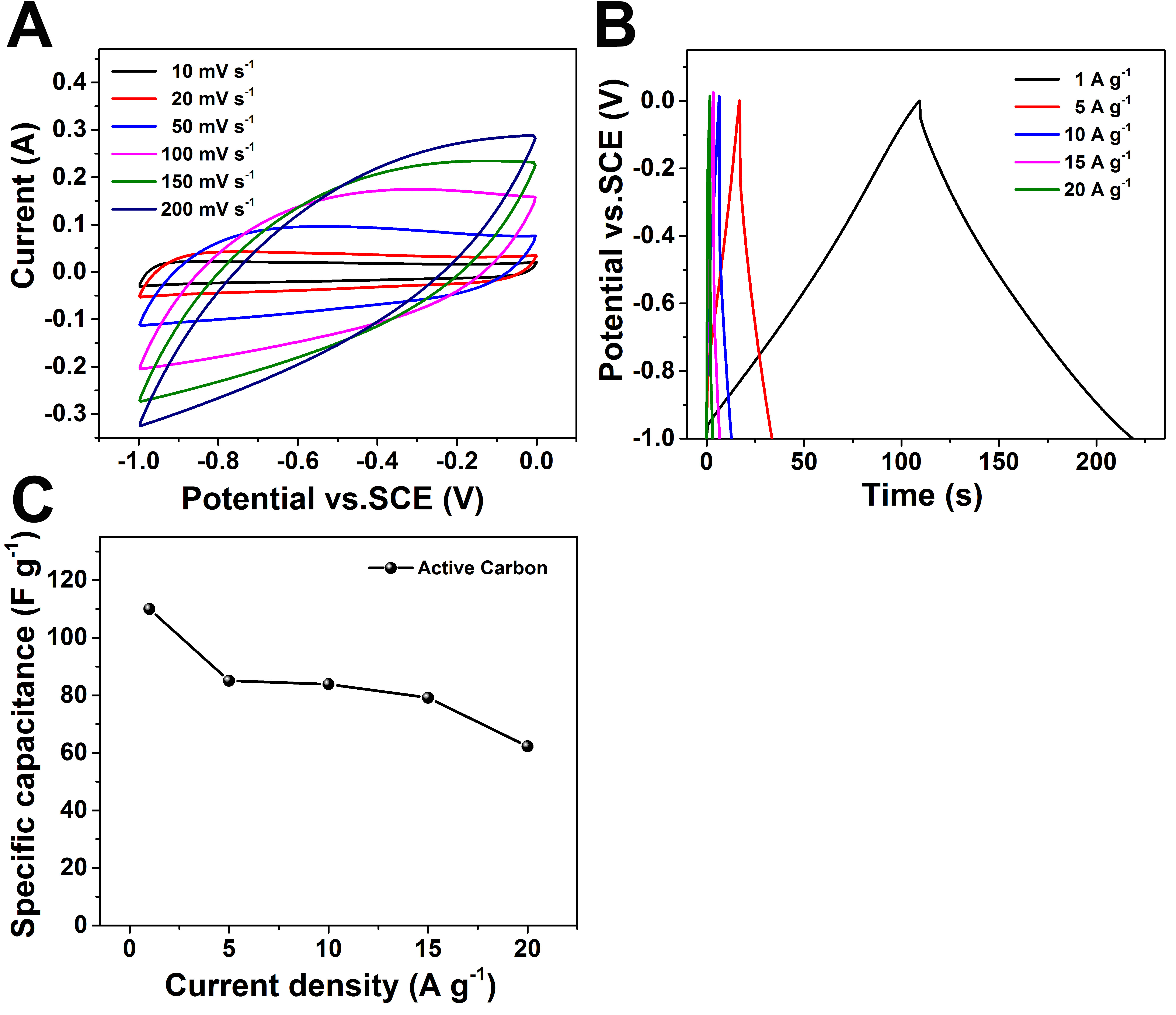


**Fig. S5** CV curves of the AC electrode at different scan rates (A), GCD curves of the AC electrode at different current densities (B), the specific capacitance change of the AC electrode at different current densities (C).


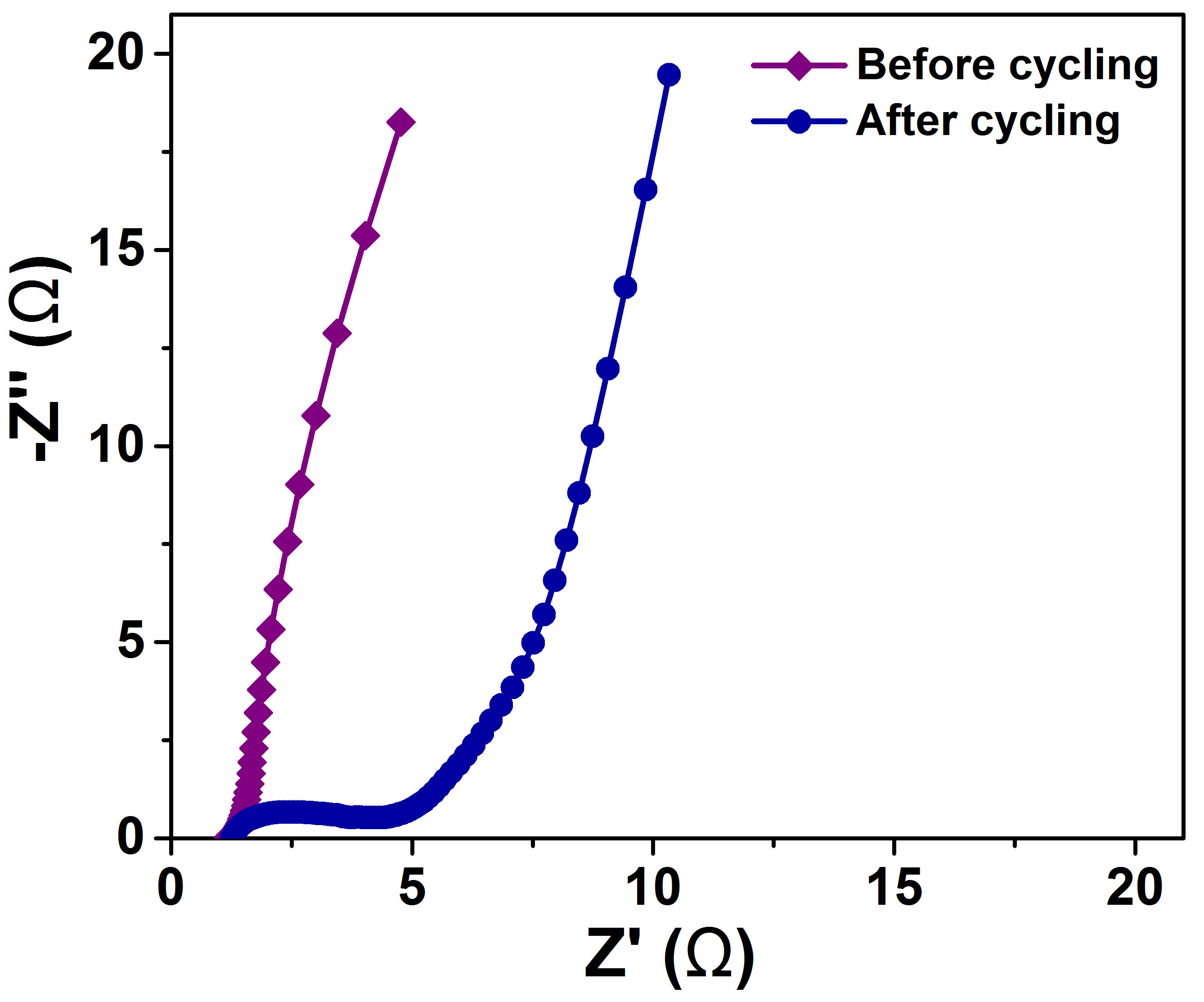


**Fig. S6** Impedance Nyquist plots of the NiCo_2_S_4_@NiMoO_4_//AC device before and after 2000 cycles.

**References**

1. Kong W, Lu CC, Zhang W, Pu J, Wang ZH. Homogeneous core-shell NiCo_2_S_4_ nanostructures supported on nickel foam for supercapacitors. J Mater Chem A, 2015;3:12452-12460.

2. Xiao K, Xia L, Liu GX, Wang SQ, Ding LX, Wang HH. Honeycomb-like NiMoO_4_ ultrathin nanosheet arrays for high-performance electrochemical energy storage. J Mater Chem A. 2015;3:6128-6135.

3. Hong W, Wang JQ, Gong PW, Sun JF, Niu LY, Yang ZG, Wang ZF, Yang SR. Rational construction of three dimensional hybrid Co_3_O_4_@NiMoO_4_ nanosheets array for energy storage application. J Power Sources, 2014;270:516-525.

4. Pu J, Wang TT, Wang HY, Tong Y, Lu CC, Kong W, Wang ZH. Direct growth of NiCo_2_S_4_ nanotube arrays on nickel foam as high-performance binder-free electrodes for supercapacitors. ChemPlusChem, 2014;79:577-583.
